# Regulation of exosome secretion by cellular retinoic acid binding protein 1 contributes to systemic anti-inflammation

**DOI:** 10.1186/s12964-021-00751-w

**Published:** 2021-06-30

**Authors:** Yi-Wei Lin, Jennifer Nhieu, Chin-Wen Wei, Yu-Lung Lin, Hiroyuki Kagechika, Li-Na Wei

**Affiliations:** 1 Department of Pharmacology, University of Minnesota, 6-120 Jackson Hall, 321 Church St. SE, Minneapolis, MN 55455 USA; 2 Institute of Biomaterials and, Bioengineering, Tokyo Medical and Dental University (TMDU), 2-3-10 Kanda-Surugadai, Chiyoda-ku, Tokyo, 01-0062 Japan

**Keywords:** Exosome, Retinoic acid, Crabp1, RIP140, Neuron, Macrophage, Inflammation

## Abstract

**Background:**

Intercellular communications are important for maintaining normal physiological processes. An important intercellular communication is mediated by the exchange of membrane-enclosed extracellular vesicles. Among various vesicles, exosomes can be detected in a wide variety of biological systems, but the regulation and biological implication of exosome secretion/uptake remains largely unclear.

**Methods:**

Cellular retinoic acid (RA) binding protein 1 (Crabp1) knockout (CKO) mice were used for in vivo studies. Extracellular exosomes were monitored in CKO mice and relevant cell cultures including embryonic stem cell (CJ7), macrophage (Raw 264.7) and hippocampal cell (HT22) using Western blot and flow cytometry. Receptor Interacting Protein 140 (RIP140) was depleted by Crispr/Cas9-mediated gene editing. Anti-inflammatory maker was analyzed using qRT-PCR. Clinical relevance was accessed by mining multiple clinical datasets.

**Results:**

This study uncovers Crabp1 as a negative regulator of exosome secretion from neurons. Specifically, RIP140, a pro-inflammatory regulator, can be transferred from neurons, via Crabp1-regulated exosome secretion, into macrophages to promote their inflammatory polarization. Consistently, CKO mice, defected in the negative control of exosome secretion, have significantly elevated RIP140-containing exosomes in their blood and cerebrospinal fluid, and exhibit an increased vulnerability to systemic inflammation. Clinical relevance of this pathway is supported by patients’ data of multiple inflammatory diseases. Further, the action of Crabp1 in regulating exosome secretion involves its ligand and is mediated by its downstream target, the MAPK signaling pathway.

**Conclusions:**

This study presents the first evidence for the regulation of exosome secretion, which mediates intercellular communication, by RA-Crabp1 signaling. This novel mechanism can contribute to the control of systemic inflammation by transferring an inflammatory regulator, RIP140, between cells. This represents a new mechanism of vitamin A action that can modulate the homeostasis of system-wide innate immunity without involving gene regulation.
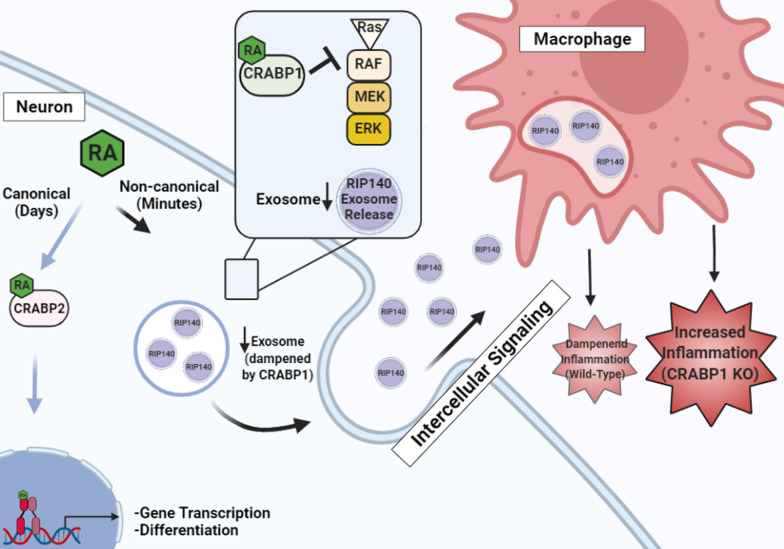

**Video Abstract**

**Supplementary Information:**

The online version contains supplementary material available at 10.1186/s12964-021-00751-w.

## Background

Intercellular communications via secreting and receiving signals are important for maintaining normal physiological processes [1, 2]. Intercellular communications can occur over a long distance such as hormonal regulation of different organ systems, or a short distance such as signaling across gap junctions between neighboring cells. The signaling molecules can be RNAs, proteins/peptides, ions, amino acids or chemicals that are usually transferred via receptors/binding proteins or membrane-enclosed extracellular vesicles [3, 4]. Among various secreted vesicles, exosomes are increasingly recognized in a wide range of biological systems; but specific physiological or pathological stimuli or regulatory pathways for exosome secretion/action remain poorly understood.

Exosomes range from 30 to 100 nm in diameter, and are generated from endosome-originated multivesicular bodies (MVBs) [4]. They are released mainly through the fusion of MVBs with plasma membrane. In the brain, exosomes can contribute to intercellular communication within the brain [5, 6], and can be involved in neurogenesis, stress responses, immune responses and synaptic plasticity [7, 8]. Since exosomes can cross blood–brain-barrier (BBB), they may also participate in certain central-peripheral communications. While immune cells of the brain such as microglia can modulate neural functions by secreting exosomes [9, 10], whether neurons can secrete exosomes to modulate immune cells is much less investigated. This study reports an example of intercellular communication initiated from neuron, via secreting exosomes, to modulate microglia/macrophage functions.

Retinoic acid (RA) is the principal active ingredient of vitamin A and essential for numerous physiological processes [11, 12]. RA is best known to exert its activities through binding nuclear RA receptors (RARs), which elicits delayed, long term effects by altering gene expression, known as the canonical signaling of RA [13]. It is known that RA can also directly elicit activities to modulate cytosolic signaling [14–20], mediated by a specific, cytosolic RA signaling protein called Cellular retinoic acid-binding protein 1 (Crabp1) [21]. Our previous studies have shown that Crabp1 levels are altered in certain pathological conditions such as under neuronal stress or neuroinflammation [16, 22], and in metabolic diseases and heart failure [23], suggesting a relevance to diseases. This current study reports a new functional role of Crabp1, that it regulates intercellular communication to impact on system wide homeostasis by modulating exosome secretion.

Receptor interacting protein 140 (RIP140) is a versatile transcription co-regulator for a wide-spectrum of transcription factors [24, 25]. RIP140 can also be exported to the cytoplasm to regulate cytosolic events, including suppressing glucose transporter 4/adiponectin vesicle trafficking in adipocytes [26, 27], and attenuating calcium release in neurons [28]. In immune cells such as macrophage, RIP140 modulates macrophage polarization by acting as a co-activator of NFκB in the nuclei to promote inflammatory response [29]. Subsequently, RIP140 is transported to the cytoplasm and becomes a phosphatase inhibitor to impair cytosolic STAT6 signaling, thereby dampening anti-inflammatory potential, and further sustaining inflammatory potential of these macrophages [30]. Therefore, for macrophages, RIP140 generally serves to enforce/maintain the inflammatory (dampen anti-inflammatory) status of the innate immune system [29–32]. This current study reports a novel mechanism of RIP140 action carried by exosomes, which contribute to system-wide inflammation.

In the following, we present data showing the regulation of intercellular, neuron-macrophage, and communication via exosomes by RA-Crabp1, which represents a novel mechanism of vitamin A action that rapidly modulates system-wide propagation of specific signals to maintain homeostasis.

## Method

### Materials and Methods

#### Animal experiments

8–12 weeks-old wild type and *Crabp1* knockout (CKO) male C57Bl/6 mice were utilized in this study. Mice were maintained in the animal facility of University of Minnesota. CKO mice were obtained and maintained as previous described [16]. Animals were euthanized by CO_2_ before samples collection. All studies were conducted according to the NIH guidelines and approved by the University of Minnesota Institutional Animal Care and Use Committee.

#### Cell Culture

CJ7 mouse embryonic stem cells were maintained as described [16]. Mouse macrophage cell line Raw 264.7 and mouse hippocampal cell line HT22 were maintained in DMEM (Gibco #11965) supplemented with 10% FBS, 1% penicillin, and 1% streptomycin. Cell numbers were counted by Invitrogen Countless II FL according to manufacturer’s instruction. Cell culture medium were collected and concentrated by 10 K Amicon Centrifugal filters (Millipore UFC9010) for further analysis.

#### Chemicals

Retinoic acid (RA, R2625), AGN 193109 (AGN, SML2034), actinomycin D (ACD, A9415), cycloheximide (CHX, C7698), and UO126 (19–147) were obtained from Millipore-Sigma. Compound 3 (C3) is known as 3-(2-(4-Chloro-N-methylbenzamido)phenyl)propanoic acid and compound 4 (C4) as (E)-3-(2-(N-Methyl-4-(methylthio) benzamido) phenyl)propenoic acid as previous described [15].

#### Plasmids

Crispr-RIP140 knock-in plasmids were generated by Biocytogen. Guide RNA sequence is 5′-GGCTTGGCTCTGATGTGCATC-3′ and RIP140 recombinant template sequence is available upon request.

#### RNA isolation and quantitative real time PCR

RNA was isolated with Trizol and converted to cDNA with High-Capacity cDNA Reverse Transcription Kit. Quantitative real-time PCR (qPCR) was performed with Maxima SYBR Green qPCR Master Mixes (Thermo Scientific) as described. Primers for Arg1 (QT00134288) were purchased from Qiagen. Each analysis was performed triplicate and normalized to β–actin.

#### Western Blotting and Immunoprecipitation

Cells were lysed and intracellular protein samples were prepared as described [31]. For immunoprecipitation, concentrated cell culture medium were precipitated with specific antibodies in Co-IP buffer. Cell lysate and precipitated samples were separated by 8–15% SDS-PAGE and transferred to polyvinylidene difluoride membranes. Membranes were incubated with specific primary antibodies and followed by HRP conjugated anti-IgG secondary antibodies. Membranes were developed and visualized by ECL (Advansta, K12045) substrate and MyECL Imager (Thermo Scientific). Flotillin-1 antibody was obtained from BD Bioscience (610821); RIP140 antibody was purchased from Abcam (AB42126); CD9 (SC-13118), β-actin (SC-47778) and GFP (SC-9996) antibodies were obtained from Santa Cruz.

#### CSF Isolation

16 weeks old WT and CKO mice were used in this experiment. The CSF collection was performed as described previously protocol with slight modifications [46]. Briefly, after anesthetizing the mice with CO_2_, the skin and musculature was displaced until the meninges on top of the cisterna magna were exposed. The CSF was immediately collected from the cisterna magna using the custom-made calibrated micropipette (Drummond Scientific, Cat. #2-000-050). Clear CSF were collected from 3 mice and pooled together. Blood‐contaminated samples were not used. CSF samples were stored in − 20 °C until use.

#### Immunohistochemistry

Brown adipose tissue samples were collected after mice were euthanized. Tissue samples were fixed, embedded in paraffin, sectioned and stained with hematoxylin and eosin. Images were taken using a Zeiss microscope (Axioplan 2 Upright).

#### Exosomes isolation and flow cytometry

Exosomes were isolated from PS Capture™ Exosome Flow Cytometry Kit (FUJIFILM Wako Chemicals) according to the manufacturer’s instructions. In brief, CSF or plasma were incubated with exosome binding enhancer and exosome capture beads at room temperature for 1 h. Exosomes were washed and resuspended in wash buffer for immunostaining. For RIP140 staining, exosome surface marker CD9 (Biolegend, clone: MZ3) was first labeled at room temperature for 1 h. Exosomes were later fixed and permeabilized with fixation/permeabilization solution kit (BD Biosciences, #554714). Briefly, exosomes were fixed in fixation/permeabilization solution at room temperature for 30 min and subsequently permeabilized with 1× permeabilization buffer and centrifuge at 400G for 10 min twice. After blocking with 100 μl 2% BSA, anti-mouse CD16/32 (BD Biosciences, clone: 2.4G2) in 1× permeabilization buffer at room temperature for 15 min, 100 μl RIP140 antibody (Abcam, ab42126) or isotype control (Abcam, ab37415) were directly added into the mixture and further incubated at room temperature for 1 h. Exosomes were later stained with FITC-conjugated secondary antibody (BD Biosciences, #554020) for 40 min on ice, then washed and resuspended in FACS buffer and analyzed by BD™ LSR II flow cytometry and analyzed by the FlowJo® software.

#### Human Data Mining

A systematic literature search revealed a study performed by Satoh et al. in which human grey and white matter samples from multiple sclerosis (MS) patients were analyzed for matter-specific differential gene expression [47]. Differential analysis was performed by Trapnell et al. using Cufflinks2.1.1 software (cufflinks.cbcb.umd.edu) [48]. Fold-change was displayed in Fig. 3d and calculated by dividing raw fragments per kilobase of exon model per million reads mapped (FKPM) units for MS over Healthy Control FKPM units (MS/Healthy).

A systemic search of the OpenTargets database (https://genetics.opentargets.org) identified differential *Crabp1* gene expression in the following diseases: Cutaneous Lupus Erythematosus, Crohn’s Disease, Vitiligo, and Psoriasis. Fold change was reported from the output generated from the OpenTargets database. For Lupus, vitiligo and psoriasis differential expression analysis was performed using the R package LIMMA v.3.28.1 by Scholtissek et al. Regazzetti et al. and Jabbari et al. respectively [35, 37, 38]. For Crohn’s disease differential expression analysis was performed using DeSeq2 v. 1.10.1 [36]. Reported *p* values are Benjamin and Hochberg corrected with a false detection rate of 0.05 [49]. Experimental details and analyses for each disease were archived in the EMBL-EBI Expression Atlas Data Repository (https://www.ebi.ac.uk/gxa/home) with the following accession IDs: Cutaneous Lupus Erythematosus (E-MTAB-5542), Crohn’s Disease (E-GEOD-57945), Vitiligo (E-GEOD-65127) and Psoriasis (E-GEOD-52471).

## Results

**Fig. 1:**
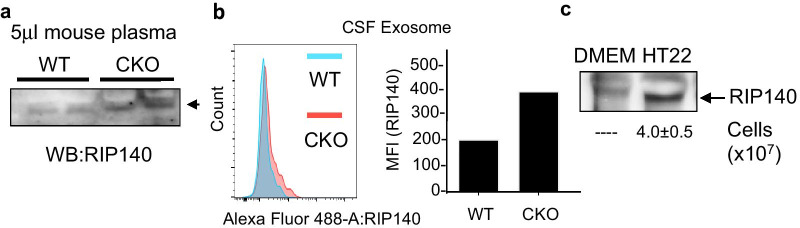
RIP140 detected in extracellular fluid/exosomes. **a** Western blot analyses of 5 µl plasma from wild-type (WT) and CRABP1 knockout (CKO) mice. **b** FACS analyses of RIP140-containing exosomes collected from cerebrospinal fluid (CSF) of WT and CKO mice. Left panel: histogram, right panel: Mean Fluorescence Intensity (MFI). **c** Western blot analyses of media collected from control (medium only) and HT22 cell culture

Our previous studies have established that RIP140, a nuclear transcription co-regulator, can be transported to the cytoplasm to augment specific cytosolic signaling pathways. It was very interesting that, in our preliminary experiments, we also detected RIP140 in mouse plasma (Additional file 1: Fig. S1a). Careful studies comparing Crabp1 knockout (CKO) and wild type (WT) mice revealed that the plasma RIP140 level was higher in CKO mice as compared to wild type mice (Fig. 1a). In addition to plasma, RIP140-containing exosomes could also be isolated from cerebrospinal fluid (CSF). Importantly, CSF RIP140 levels were also higher in CKO than WT mice (Fig. 1b). These data suggested that Crabp1 could play a regulatory role in the secretion of RIP140 exosomes, which was further validated in molecular experiments (see later). To first demonstrate that the extracellular RIP140 was secreted from specific cells, we exploited a known RIP140-expressing cell culture system, the hippocampal neuron cell line HT22. This system allowed monitoring the secretion of RIP140. Clearly, RIP140 could be detected in the culture medium of HT22 (Fig. 1c). Herein RIP140 derived from exosomes was denoted as “exo-RIP140.”

**Fig. 2:**
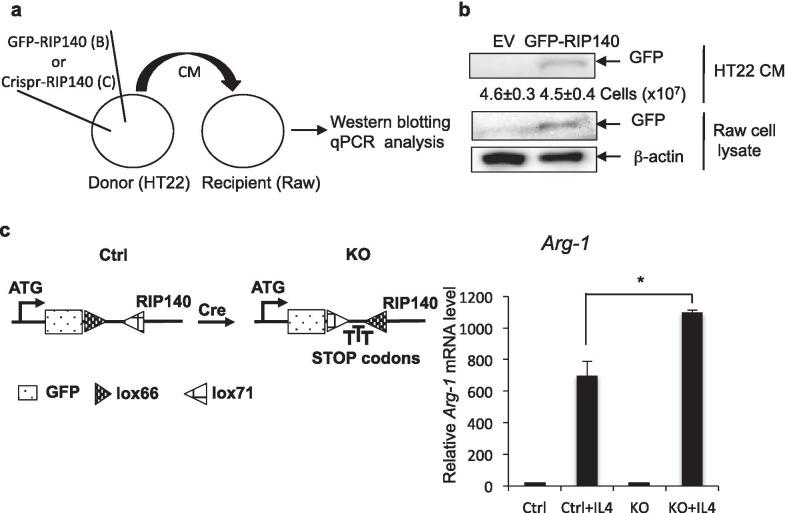
Transfer of RIP140-containing exosomes from neuron to macrophage. **a** Experimental diagram for (**b**) and (**c**). **b** Western blot analyses. GFP (representing GFP-RIP140) was detected in HT22 CM (top lane, donor cells) and receiving cell, Raw 264.7, lysate (bottom panel). **c** Left panel: Design of RIP140 editing to tag and knockout endogenous RIP140 of HT22 by Cre-recombinase. Right panel: qPCR of Arg-1 expression in Raw 264.7 cells after receiving HT22 CM. Data show 3 experimental results with Student’s t test (n = 3). *p ≤ 0.05. Error bars are represented as ± the Standard Error of the Mean (SE)

We next determined whether the secretion of exo-RIP140 from neurons was a productive process, i.e. if exo-RIP140 can be taken by specific cells to exert biological activity, thereby mediating specific intercellular communication. It has been established that an important biological activity of RIP140 is to stimulate pro-inflammatory polarization of macrophage [29–31], and that phagocytosis represents a key physiological function of macrophage/microglia. Therefore, we speculated microglia/macrophage as one type of cells receiving these neuronal exosomes, so we first determined whether exo-RIP140 of neurons could enter macrophages. In order to monitor exo-RIP140 derived from neuronal exosomes, we tagged RIP140 with GFP in donor cells (HT22) by expressing GFP-RIP140 in HT22 cells, and collected their conditioned medium (CM). The CM of HT22 was used to feed macrophage Raw 264.7 cells (Fig. 2a) and their cellular extracts were monitored on western blots. Figure 2b showed that GFP-RIP140 was detected in the CM of the donor HT22 cells as predicted (upper panel). Importantly, the HT22 derived GFP-labeled exo-RIP140 was also detected inside the receiving macrophage (lower panel). This result unambiguously demonstrated that exo-RIP140 was secreted from donors (HT22 neurons) and entered the recipients (macrophages), showing transfer/mobilization of RIP140 between different cell types via exosomes.

To determine whether exo-RIP140, upon entering macrophage, remained biologically active, we exploited a well-established system where elevating RIP140 level in macrophages triggers their inflammatory polarization and dampens anti-inflammation. To conduct this experiment in a physiologically relevant context, i.e. monitoring exo-RIP140 specifically derived from endogenous RIP140 of donor neurons, we employed Crispr-cas9 to edit the endogenous RIP140 locus of HT22. Specifically we edited both alleles of endogenous RIP140 locus in HT22 by introducing GFP to tag endogenous RIP140 and adding early termination codons to delete (knock out) its coding region (Fig. 2c left), generating RIP140 KO HT22 cells. By performing transfer experiments similar to that described in Fig. 2a, we compared the effect of exo-RIP140 from wild type control (Ctrl) and RIP140 knockout (KO) HT22 (Fig. 2c right). We found that, for macrophage incubated with the CM of RIP140 KO HT22 (bars 3 and 4), their anti-inflammatory potential (induced by IL4, and marked by the expression level of Arg-1) (bar 4) was significantly elevated when compared to those cells incubated with the CM of ctrl HT22 (bar 2) (Fig. 2c, right panel), indicating that the IL4-induced anti-inflammatory status of receiving macrophages was elevated when the donor’s RIP140 (inflammatory) was knocked out. This result showed that exo-RIP140 remained biologically active upon entering macrophages. This experiment clearly showed that, neuronal exo-RIP140 could enter recipient cells (macrophages) and remain biologically active to affect their polarization. These results suggest a potentially global function of RIP140 in the whole animal by exosome-mediated transfer/mobilization to augment system-wide innate immune status. Since exo-RIP140 was significantly elevated in CKO mice (Fig. 1), we predicted that the CKO mice might exhibit systemic vulnerability to inflammatory stimuli (see following).

**Fig. 3:**
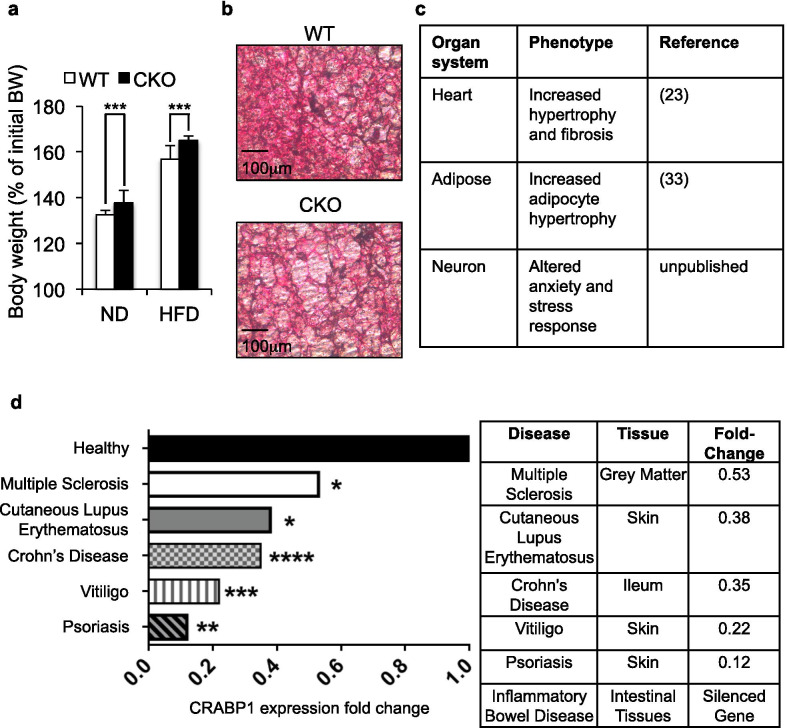
CKO mice exhibit a pro-inflammatory phenotype. **a** The percentage of initial body weight change, after 10 weeks normal (ND) and high-fat diet (HFD) feeding. n = 12 in WT-ND; n = 6 in WT-HFD, CKO-ND and CKO-HFD. Results are presented as mean ± SE ***p < 0.001 compared to WT. **b** H&E staining of brown adipose tissue of WT and CKO mice. **c** CKO mouse inflammatory phenotype observed in the heart, adipose tissue and brain. **d** Human CRABP1 expression data. Reduced CRABP1 expression in various inflammatory disease conditions. Results are presented as *p value ≤ 0.05; **p value ≤ 0.01; ***p value ≤ 0.001; ****p value ≤ 0.0001. [34–38]

We systemically evaluated inflammation-related phenotypes of CKO mice as shown in Fig. 3a–c, including data reported earlier. These include increased high fat diet-induced obesity (Fig. 3a), and adipose expansion and hypertrophy (Fig. 3b), as well as insulin resistance [33], hyper-sensitivity to isoproterenol-induced cardiac hypertrophy that mimics inflammation [23], and altered anxiety and stress response (unpublished) (Fig. 3c). All these data support the notion of increased vulnerability of CKO mice in response to inflammatory stimuli on a system level. To examine whether this notion, that Crabp1 modulates the inflammatory status in animals, was clinically relevant, we performed clinical data mining of available patients data. It appeared that Crabp1 expression was dramatically and significantly reduced in multiple human inflammatory diseases including multiple sclerosis, lupus, inflammatory bowel diseases, vitiligo and psoriasis [34–38] (Fig. 3d). All these results together show that Crabp1 plays a role in regulating system-wide inflammatory response, at least partially, via modulating the mobilization of a pro-inflammatory regulator RIP140 through secreted exosomes.

**Fig. 4:**
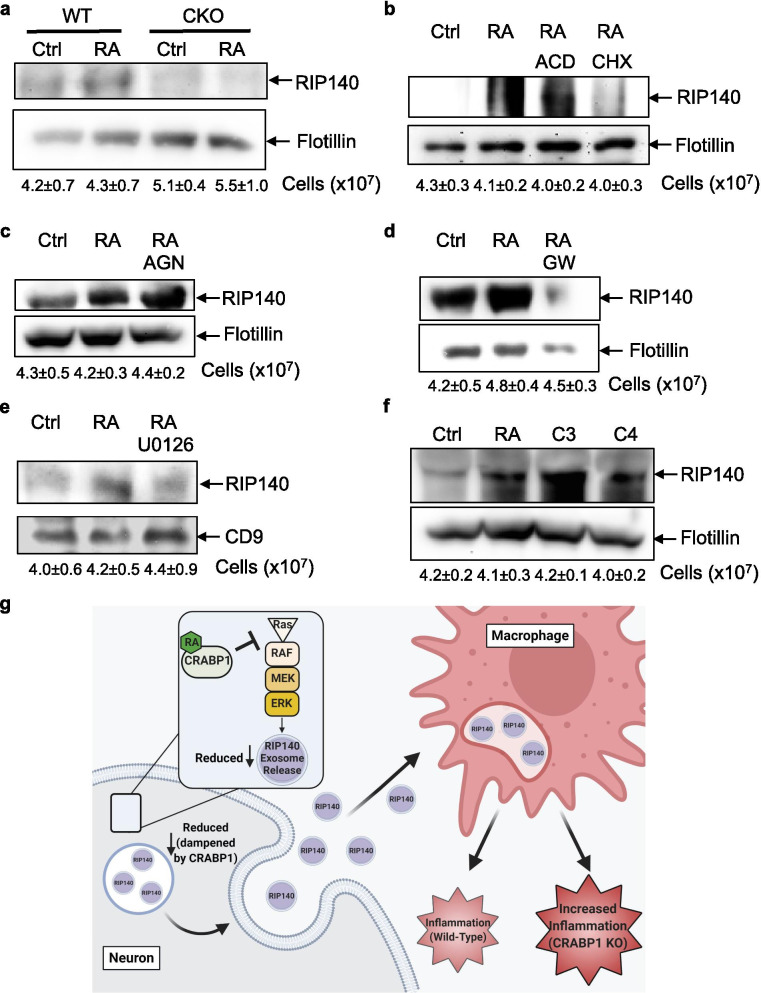
RA-Crabp1 modulates exosome secretion via the MAPK kinase pathway. **a** Western blot analyses using antibodies against RIP140 and flotillin to examine the medium of ESC cultured with RA (100 nM) or control (Ctrl). **b** Western blot analyses of HT22 culture medium, in cultures pre-treated with 1 µg/ml ACD or 10 µg/ml CHX 1 h, and followed by 100 nM RA treatment. ACD: actinomycin D, CHX: cycloheximide. **c** Western blot analyses of HT22 culture medium, in cultures pretreated with 100 nM AGN 1 h, and followed by 100 nM RA treatment. AGN: AGN 193109. **d** Western blot analyses of HT22 culture medium, in cultures pretreated with 10 µM GW 1 h, and followed by 100 nM RA treatment. GW: GW4869. **e** Western blot analyses, using antibodies against RIP140 and CD9 of medium collected from HT22 culture pretreated with 10 µM U0126 for 1 h, followed by 100 nM RA treatment. **f** Western blot analyses of medium collected from HT22 culture treated with 100 nM RA, C3 or C4. C3: compound 3; C4: compound 4, chemical names are provided in materials and methods. **g** A model for RA-Crabp1 action in negatively regulating RIP140-containing neuronal exosome secretion to modulate inflammation. Created with https://BioRender.com

To examine the underlying mechanism of the action of Crabp1 in regulating exosome secretion, we employed an in vitro system where both Crabp1 and RIP140 were endogenously expressed and CKO was readily available, an embryonic stem cell (ESC) line from CKO mice [16]. Since Crabp1 has a specific ligand, RA, we first examined if RA could affect the secretion of RIP140 from ESC. As shown in Fig. 4a, RA enhanced RIP140 secretion from WT ESC maintained in cultures without additional growth factors; importantly, this was abolished in CKO cells. Thus, RA acts on Crabp1 to regulate exosome secretion. To rule out potential effects involving gene transcription mediated by RARs, we used a transcription inhibitor, actinomycin D in the experiments. Figure 4b showed that exo-RIP140 secretion was clearly independent of gene transcription. As a control, it involved protein expression (i.e., inhibition by cycloheximide). To further validate this RAR-independent activity of RA, we used a pan-RAR antagonist, AGN 193109, to block all RAR-mediated nuclear events in the experiment. As shown in Fig. 4c, AGN 193109 could not inhibit RA-induced exo-RIP140 secretion, further supporting a Crabp1-dependent activity of RA in regulating exo-RIP140 secretion. Indeed, an exosome secretion blocker (GW4869) dramatically inhibited the secretion of exo-RIP140, validating that RA/Crabp1-regulated RIP140 secretion was via exosomes (Fig. 4d).

Two signaling pathways could be directly targeted by Crabp1, the MAPK and CaMKII signaling pathways [19]. Interestingly, RIP140 exosome secretion was blocked by MAPK inhibitor U0126 (Fig. 4e), but not CaMKII inhibitor (not shown). Furthermore, two previously identified selective ligands of Crabp1 [15], C3 and C4, were also able to act on Crabp1 to regulate exo-RIP140 secretion as effectively as RA (Fig. 4f). These results validate that exo-RIP140 secretion indeed involves RA-Crabp1, and that this is mediated by a specific Crabp1 target, the MAPK pathway.

It is worthy of notion that, in the culture system where no additional growth factors were provided, RA/Crabp1 could activate MAPK signaling (Raf-Mek-Erk) via binding Crabp1 to block the initiating Raf kinase [14]. In a typical growth factor-activated MAPK signaling, it is most robustly activated by Ras. In this context, RA-Crabp1 acted to compete with Ras for Raf activation, thereby dampening Raf-Mek-Erk signaling in a normal physiological context [17]. Therefore, RA-Crabp1 is a negative regulator of growth factor-elicited MAPK signaling. CKO mice are deficient in this negative regulation of MAPK signaling, and their exosome secretion would be elevated as compared to WT. This is supported by the experimental data (Figs. 1, 2 and 3).

We proposed a model (Fig. 4g) for the action of RA-Crabp1 in regulating exosome secretion, which contributes to system-wide modulation of physiological processes including inflammatory response. In general, growth factors or mitogen signals are the principal triggers of the MAPK signaling pathway [39] to stimulate exosome secretion [40–42], including RIP140-containing exosome. This would maintain a certain level of system-wide propagation of inflammatory potential. RA-Crabp1, via dampening the MAPK signaling, would provide a negative modulating mechanism in order to tone down global inflammation when needed. If Crabp1 is deleted or defected, as in CKO mice that lack this negative counter-balance mechanism, the vulnerability to inflammatory stimuli would be increased.

## Discussion

Of most interest in this study is the identification of RA-Crabp1 as one physiological, negative regulator of exosome secretion. The study also reports, for the first time, that the inflammatory coregulator RIP140 can be transferred from neurons to macrophages, via secreted exosomes, which contributes to system wide propagation of inflammatory signal. By negatively regulating exosome secretion, RA-Crabp1 dampens the system-wide propagation of inflammatory signals. Therefore, CKO mice are prone to inflammatory stimulation. This study also determines the mechanism of Crabp1 action in regulating exosome secretion, which is mediated by its direct target, the MAPK signaling pathway. These results also suggest that vitamin A can be a physiological regulator of inter-cellular communications, such as by regulating exosome secretion. This is mediated by its specific cytosolic signaling protein, Crabp1.

Under a normal condition, mitogen/growth factors provide stimuli for Ras/Raf/Mek/Erk signaling for normal exosome secretion [40–43]. RA-Crabp1, under a healthy vitamin A status, plays a negative regulatory role to dampen exosome (and exo-RIP140) secretion, thereby maintaining inflammatory homeostasis. The CKO mice are deficient in this systemic inflammation-dampening mechanism; therefore they are more vulnerable to inflammatory stimuli in multiple organ systems.

The pro-inflammatory phenotype of CKO mice is consistent with available clinical data, that Crabp1 expression is significantly reduced in multiple inflammatory diseases. RA is also known to play roles in regulating the immune system [11, 44, 45], attributed to its activities via RAR-mediated gene regulation. This involves, mainly, cell differentiation and the maturation of a healthy immune system. Our current study adds an additional level of complexity in the mechanisms of vitamin A action, attributable to the modulation of systemic homeostasis of physiological processes including inflammation/anti-inflammation in the adults, via regulating exosome secretion to facilitate rapid intercellular communication. Presumably, this particular activity of RA can provide a timely (before RA eliciting its canonical RAR-dependent activities) mechanism to modulate the dynamics of system-wide physiological processes such as innate immunity. How this interacts with the physiological processes regulated by RAR-dependent genomic activities of RA remains to be studied. Nevertheless, it is tempting to speculate that this additional mechanism will greatly enhance the capacity and flexibility of an organism in the face of certain urgent situations, such as when a timely response or rapid propagation of specific signals is needed. It would also be important to investigate other contents of these exosomes, and how this new activity of RA-Crabp1 may be affected by the vitamin A status.

With regards to the relevance of our experimental system, we have exploited hippocampus neural cells as the source of RIP140-exosomes because these neurons naturally express RIP140 and Crabp1. In this experimental system, both the content (RIP140) and the regulator (Crabp1) of exosomes are endogenously expressed, providing a more physiologically relevant context for experimentation. In selecting specific target cells for this study, we have utilized macrophages because it is an immune cell type where RIP140’s pro-inflammatory effect has been established. However, it would be interesting to investigate other cell types as potentially additional source of exosomes, where Crabp1 and RIP140 are both expressed. This will clarify whether this new mechanism could be generalized. In addition, it would be of clinical interest to examine if exo-RIP140 in the circulation (such as blood) could provide a biomarker of innate immune status, and if exo-RIP140 could provide a new therapeutic target. Finally, additional contents of exosomes secreted from these neurons remain to be investigated.

## Conclusions

Our current study demonstrates regulation of neuronal exosome secretion by a specific physiological signal, via RA-Crabp1-MAPK signaling. The study also presents the first evidence for intercellular transfer of an important inflammatory regulatory molecule, RIP140, to modulate innate immunity and systemic inflammation. Clinical relevance of this new regulatory pathway, involving Crabp1, is supported by patients’ data of multiple human inflammatory diseases. Of clinical interest is to validate whether exo-RIP140 in human circulation (such as blood) could provide a biomarker of patients’ inflammatory status, and if this might present a new therapeutic target in managing diseases related to inflammation.

## Supplementary Information

**Additional file 1: Fig. S1.** **a** FACS analysis of mouse plasma using RIP140 antibody. **b** FACS analysis of mouse CSF exosomes using RIP140 antibody.

## Data Availability

The data generated are included in the manuscript and supplementary data.

## Abbreviations

RA: Retinoic acid
CRABP1: Cellular retinoic acid binding protein 1
RIP140: Receptor interacting protein 140
CKO: CRABP1 knockout
MVBs: Multivesicular bodies
BBB: Blood–brain-barrier
RAR: Retinoic acid receptors
CaMKII: Calmodulin dependent protein kinase II
MAPK: Mitogen-activated protein kinase
CSF: Cerebrospinal fluid
CM: Conditioned medium
ACD: Actinomycin D
CHX: Cycloheximide
AGN: GN 193109
GW: GW4869
