# Supplementary figures and images for: Regulation of exosome secretion by cellular retinoic acid binding protein 1 contributes to systemic anti-inflammation
Source: Cell Commun Signal. 2021 Jun 30;19:69. doi: 10.1186/s12964-021-00751-w (PMC8247179; doi:10.1186/s12964-021-00751-w)

**a**

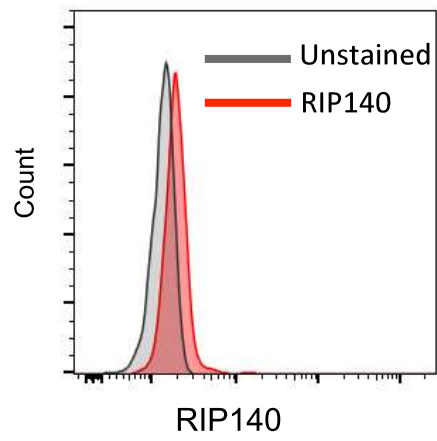

**b**

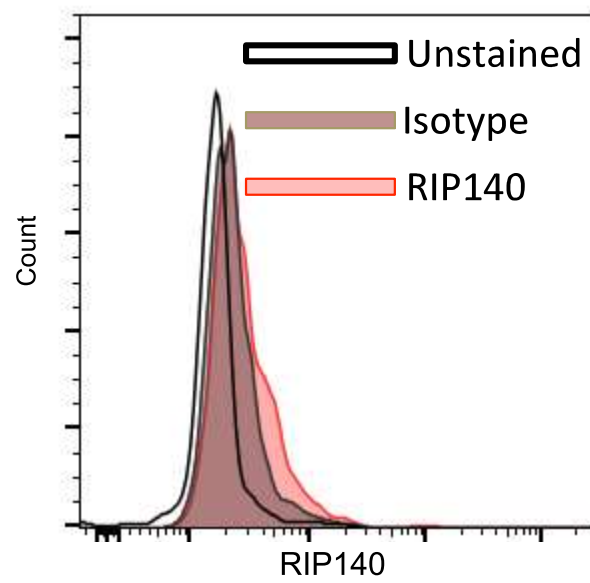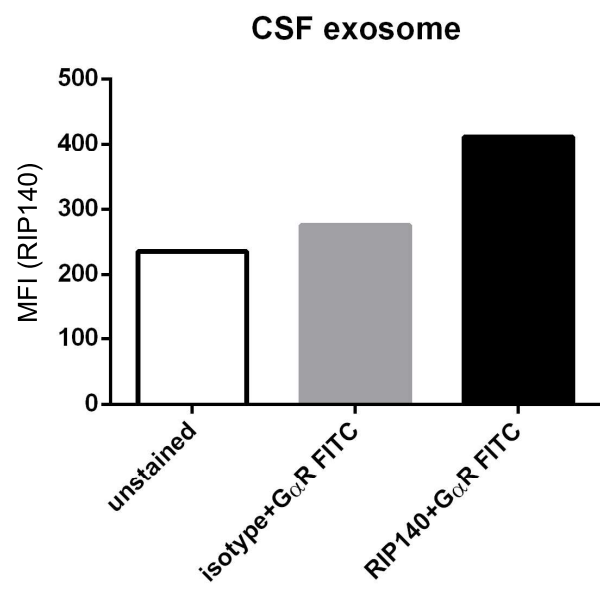

**Fig. S1. Lin et al**

Supplement: Supplementary file 2 — Additional file 1: Fig. S1. a FACS analysis of mouse plasma using RIP140 antibody. b FACS analysis of mouse CSF exosomes using RIP140 antibody. [file 12964_2021_751_MOESM2_ESM.pdf]
